# Changes in functional brain organization and behavioral correlations after rehabilitative therapy using a brain-computer interface

**DOI:** 10.3389/fneng.2014.00026

**Published:** 2014-07-15

**Authors:** Brittany M. Young, Zack Nigogosyan, Léo M. Walton, Jie Song, Veena A. Nair, Scott W. Grogan, Mitchell E. Tyler, Dorothy F. Edwards, Kristin Caldera, Justin A. Sattin, Justin C. Williams, Vivek Prabhakaran

**Affiliations:** ^1^Department of Radiology, University of Wisconsin–MadisonMadison, WI, USA; ^2^Medical Scientist Training Program, University of Wisconsin–MadisonMadison, WI, USA; ^3^Neuroscience Training Program, University of Wisconsin–MadisonMadison, WI, USA; ^4^Department of Biomedical Engineering, University of Wisconsin–MadisonMadison, WI, USA; ^5^Departments of Kinesiology and Medicine, University of Wisconsin–MadisonMadison, WI, USA; ^6^Department of Orthopedics and Rehabilitation, University of Wisconsin–MadisonMadison, WI, USA; ^7^Department of Neurology, University of Wisconsin–MadisonMadison, WI, USA

**Keywords:** brain-computer interface, stroke rehabilitation, laterality index, LI, BCI therapy, UE motor recovery, fMRI

## Abstract

This study aims to examine the changes in task-related brain activity induced by rehabilitative therapy using brain-computer interface (BCI) technologies and whether these changes are relevant to functional gains achieved through the use of these therapies. Stroke patients with persistent upper-extremity motor deficits received interventional rehabilitation therapy using a closed-loop neurofeedback BCI device (*n* = 8) or no therapy (*n* = 6). Behavioral assessments using the Stroke Impact Scale, the Action Research Arm Test (ARAT), and the Nine-Hole Peg Test (9-HPT) as well as task-based fMRI scans were conducted before, during, after, and 1 month after therapy administration or at analogous intervals in the absence of therapy. Laterality Index (LI) values during finger tapping of each hand were calculated for each time point and assessed for correlation with behavioral outcomes. Brain activity during finger tapping of each hand shifted over the course of BCI therapy, but not in the absence of therapy, to greater involvement of the non-lesioned hemisphere (and lesser involvement of the stroke-lesioned hemisphere) as measured by LI. Moreover, changes from baseline LI values during finger tapping of the impaired hand were correlated with gains in both objective and subjective behavioral measures. These findings suggest that the administration of interventional BCI therapy can induce differential changes in brain activity patterns between the lesioned and non-lesioned hemispheres and that these brain changes are associated with changes in specific motor functions.

## Introduction

Stroke remains a growing source of disability, with nearly 800,000 individuals in the United States alone experiencing a stroke each year and a projected increase of 22% in stroke prevalence by 2030 (Go et al., 2013). Despite increases in stroke incidence, stroke deaths have declined in recent years (Go et al., 2013), such that the majority of stroke patients survive their initial stroke event. Individuals in this growing population of stroke survivors are often left with persistent functional deficits. One common deficit is the acquisition of a lasting motor impairment, with up to 50% of stroke survivors suffering from some degree of hemiparesis and 26% needing assistance with activities of daily living (ADLs) 6 months post-stroke (Kelly-Hayes et al., 2003).

It has long been known that most patients experience some amount of spontaneous functional recovery shortly after stroke, with additional improvements that may be gained through the use of rehabilitative therapies in the months following the stroke event. Current guidelines note that there exists a lack of evidence to guide the optimal selection of a particular type, intensity, and amount of rehabilitative motor therapy for stroke survivors (Miller et al., 2010), and many stroke patients reach a functional plateau before complete recovery is achieved despite the use of currently available standard rehabilitative therapies. However, studies have suggested that clinically relevant plasticity and recovery potential still persist even after this plateau has been reached and that it may be possible to harness this reserve of recovery potential through the use of alternative, non-traditional therapies (Cramer, 2010) that incorporate components such as virtual reality (Orihuela-Espina et al., 2013), robot-assisted movement therapy (Lo et al., 2010; Pinter et al., 2013), and constraint-induced movement therapy (CIMT) (Johansen-Berg et al., 2002a; Moss and Nicholas, 2006; Gauthier et al., 2008; Wolf et al., 2008, 2010; Dromerick et al., 2009; Volpe et al., 2009; Lang et al., 2013).

Another of these alternative non-traditional therapies that shows promise in stimulating additional recovery in stroke patients incorporates devices that use an emerging type of technology called brain-computer interface (BCI). EEG-based BCI is a developing technology that detects neural activity and can use these signals as a means of providing real-time feedback by which users may learn to modulate their brain activity. It has been shown that people with disabilities have no greater mental workload compared to healthy controls when using BCI devices (Felton et al., 2012), making the potential for BCI therapy in stroke patients with persistent motor disabilities both appropriate and accessible. Currently, BCI technology is being incorporated into a new class of devices intended to facilitate motor recovery in stroke patients, and early studies are beginning to show potential functional benefits associated with the use of these devices (Buch et al., 2008; Daly et al., 2009; Broetz et al., 2010; Prasad et al., 2010; Caria et al., 2011; Shindo et al., 2011; Liu et al., 2012; Takahashi et al., 2012). One of the motivations behind this emerging trend is the idea that BCI technology may provide a means of promoting neuroplastic change, harnessing the recovery potential that remains once patients have reached a functional plateau with standard therapies.

There is reason to believe that therapy using BCI devices may produce changes in brain activation patterns concurrent with observed behavioral changes. Such changes have been observed using other interventional rehabilitative approaches in stroke patients (Carey et al., 2002; Gauthier et al., 2008; Richards et al., 2008), and changes in brain activity patterns after stroke have also been associated with functional gains in stroke patients who experience spontaneous recovery (Ward et al., 2003). Since BCI therapies provide real-time feedback to the user, effectively rewarding the consistent production of some patterns of brain activity relative to others in the context of an intended task, there may be observable changes in the brain activation patterns produced when attempting tasks similar to those trained with BCI therapy. While this theoretical knowledge supports the possibility of inducing changes in brain activation through BCI therapy, little is yet known about the specific patterns of brain change induced in stroke patients by therapies that incorporate BCI devices.

Brain changes can be measured by laterality index (LI)–a means of quantifying the degree to which a particular task or function is lateralized between the two hemispheres of the brain. LI can be used as a measure of functional brain organization (Kundu et al., 2013) and has been used to examine functional brain reorganization in stroke patients with motor deficits using other therapy modalities. For example, LI calculations have shown repetitive transcranial magnetic stimulation therapy to produce increased laterality toward the lesioned hemisphere in those with bilateral LI at baseline (Yamada et al., 2013). Improved function has also been observed with increases in laterality toward the lesioned hemisphere after mirror therapy (Bhasin et al., 2012) or CIMT (Johansen-Berg et al., 2002a), and recruitment of contralesional motor areas has been associated with better functional improvements after gesture therapy (Orihuela-Espina et al., 2013). Still other studies of neuroimaging after rehabilitative motor therapy using such approaches have shown functional gains in the absence of detectable differences in LI after constraint-induced movement (Kononen et al., 2012) or robot-assisted (Pinter et al., 2013) therapies. Whether these conflicting patterns of change across studies represent underlying differences in the populations studied, differential effects to various interventional therapy modalities producing the functional gains, or other confounding effects remains a question that has yet to be definitively answered.

Studies that have examined changes in brain activity after BCI combined with other therapies in these patients have found brain-behavior correlations in the context of functional connectivity and its relationship to the rate at which individuals achieved gains in motor function using a BCI-robotics treatment protocol (Varkuti et al., 2013) as well as in the association between LI changes and motor function improvements using a BCI-physiotherapy treatment protocol (Caria et al., 2011; Ramos-Murguialday et al., 2013). However, such BCI studies and analyses to date remain few and limited in the context of combined therapies. The aim of the present study was to investigate the effect of interventional therapy using a closed-loop neurofeedback BCI device intended to improve motor function in stroke patients on task-derived patterns of brain activation and to assess whether any observable changes in these activation patterns correlated to changes in behavioral outcomes. We examined subjective and objective measures of motor function and obtained fMRI scans of brain activation patterns during a finger tapping task at four different time points with and without the administration of BCI therapy. We hypothesized that changes in brain activation during tapping would be observable with the administration of BCI therapy. We also examined the relationship between changes in LI with gains in motor function, hypothesizing that changes observed in LI would correlate with changes in motor function.

## Materials and methods

### Recruitment methods and exclusion criteria

Patients with persistent upper extremity motor impairment resulting from first-ever ischemic or hemorrhagic stroke were contacted regarding study participation after being identified as appropriate for participation in the study by one or more physicians responsible for their care at the University of Wisconsin Hospital and Clinics. Exclusion criteria included concurrent diagnoses of neurodegenerative disorders (e.g., dementia), other neurological or psychiatric disorders (e.g., epilepsy, schizophrenia, substance abuse), or cognitive deficits that would preclude the ability to provide informed consent. Subjects were also excluded if they had any allergies to electrode gel, surgical tape, or metals, had undergone treatment for recent infectious diseases, had apparent lesions or inflammation of the oral mucosa, were pregnant or likely to become pregnant during the course of the study, or had any contraindications to MRI.

### Ethics statement

All subjects provided written informed consent. This study, including all measures assessed and therapies administered, was approved by the Health Sciences Institutional Review Board of the University of Wisconsin–Madison.

### Randomization and study paradigm

**Figure 1:**
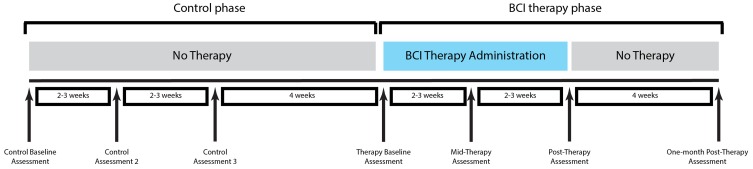
**Study paradigm**. Functional assessment using Stroke Impact Scale, Action Research Arm Test, Nine-Hole Peg Test, and neuroimaging using fMRI were performed at each assessment.

A permuted-block design accounting for gender, stroke chronicity, and severity of motor impairment was used to randomize subjects to either a BCI therapy group or a crossover control group. Subjects assigned to the BCI therapy group began participation in the BCI therapy phase of the study and received interventional rehabilitation therapy using the BCI system with functional assessment and MRI scanning at four time points: before the start of BCI therapy, at the midpoint of BCI therapy, upon completion of all BCI therapy, and 1 month following the last BCI therapy session. Subjects assigned to the crossover control group first received three additional functional assessments and MRI scans during the control phase of the study in which no BCI therapy was administered, with assessments spaced at intervals analogous to those administered during the BCI therapy phase of the study. Upon completion of the control phase of the study, subjects in the crossover control group crossed over to complete the BCI therapy phase of the study. This study paradigm is summarized in Figure 1.

### Functional assessments

Subjects' motor function of the impaired arm was assessed using a neuropsychological battery which included both subjective and objective measures. These measures included the Stroke Impact Scale 3.0 (SIS), which is a self-report measure used to assess the ability of a stroke survivor to perform in the domains of strength, hand function, activities of daily living, mobility, communication, emotion, memory and thinking, and participation (Duncan et al., 1999; Carod-Artal et al., 2008). For this study, scores from the Hand Function domain of the SIS were of particular interest, as the BCI therapy administered is designed to promote motor rehabilitation of the upper extremity. Also assessed were subject performance on the Action Research Arm Test (ARAT), which is a standardized series of scored movements designed to evaluate upper extremity motor function in the domains of grip, grasp, strength, and gross movement (Carroll, 1965; Lang et al., 2006), and the Nine-Hole Peg Test (9-HPT), which is a timed task in which the subject attempts to first place then remove each of 9 pegs from a pegboard using only one hand (Beebe and Lang, 2009). In keeping with standard scoring practices, raw scores for the hand function domain of the SIS were transformed to adjust for the lowest possible raw score and the possible raw score range. Scores for the ARAT were reported as the total points scored when using the impaired hand, and scores for 9-HPT were taken as an average of two timed trials using the impaired hand. At each of the visits for neuropsychological evaluation, anatomical and functional MRI scans were also obtained for each subject.

### Intervention

**Figure 2:**
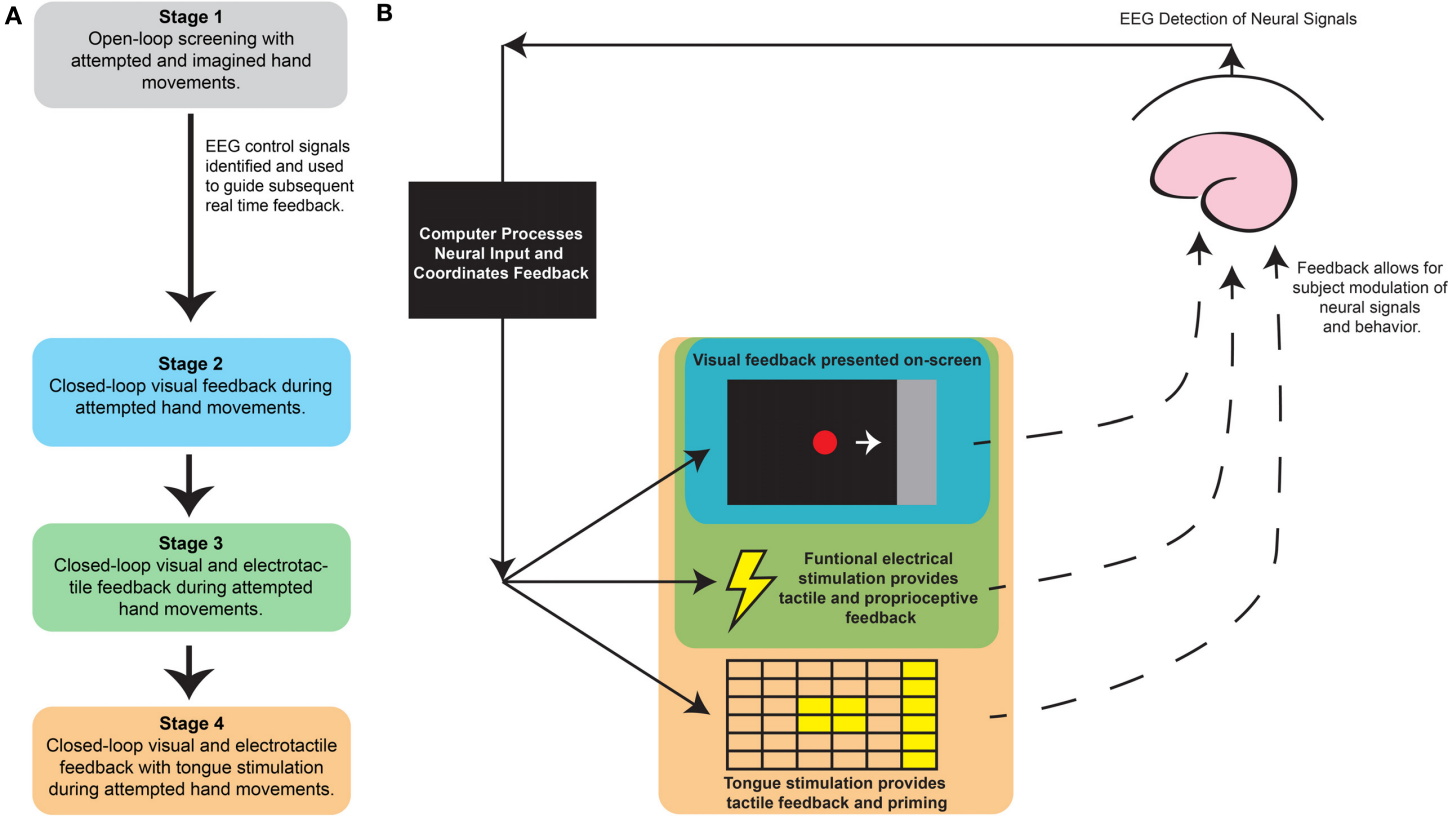
**Brain-Computer Interface interventional therapy schematic and session outline. (A)** Sequence of BCI interventional therapy session with **(B)** SmartFES device setup with color coding indicating feedback stimuli for the relevant session stages. BCI, Brain-Computer Interface.

All subjects were administered at least 9 and up to 15 two-hour sessions of interventional BCI therapy using SmartFES, a closed-loop neurofeedback device incorporating multi-modal feedback triggered by EEG. This multi-modal feedback included visual feedback, functional electrical stimulation (FES), and tongue stimulation (TS). A diagram of the device and the sequential stages of each interventional therapy session are provided in Figure 2. A detailed description of the procedures followed during each session is provided in the supplementary methods. These sessions took place over a period of up to 6 weeks with two to three therapy sessions per week.

### fMRI paradigm

Subjects performed a block-design sequential finger tapping task during fMRI scans that consisted of alternating 20-s blocks of tapping vs. rest. Subjects were cued to rest or to tap the fingers of one hand sequentially on a button box, using either visual or tactile (for visually impaired subjects) cues.

Subjects were asked to undergo two fMRI scans using this paradigm—once when tapping with the impaired hand (passive tapping if unable to generate sufficient tappings independently) and again when tapping with the unimpaired hand. Subjects were instructed to hold their heads still throughout the scans, and sufficient padding was provided to discourage head movement.

### Image acquisition and processing

MRI data was collected on one of two 3 Tesla GE MR750 scanners equipped with high-speed gradients (Sigma GE Healthcare, Milwaukee Wisconsin) using an 8-channel head coil. Padding around the subjects' heads was used to help minimize movements during scans. Functional scans were run using a T2^*^-weighted gradient-echo echo planar imaging (EPI) pulse sequence sensitive to BOLD contrast. Technical parameters used to acquire these EPI scans are as follows: field of view 224 mm, matrix 64 × 64, TR 2600 ms, TE 22 ms, flip angle 60°, and 40 axial plane slices of 3.5 mm thickness with 3.5 mm spacing between slices. During each fMRI scan, 70 sequential whole-brain acquisitions were recorded. These scanning parameters allowed for complete mapping of the cortex. A T1-weighted high-resolution anatomical image was also obtained for each subject using a BRAVO FSPGR pulse sequence during each MRI scanning session. Technical parameters used to acquire these scans are: field of view 256 mm, matrix 256 × 256, TR 8.16 ms, TE 3.18 ms, flip angle 12°, and 156 axial plane slices of 1 mm thickness with 1 mm spacing between slices.

All pre- and post-processing of MRI data was performed using the AFNI software package (Cox, 1996). The first four volumes of each functional sequence were discarded to allow for signal stabilization. EPI data sets were motion corrected and then spatially smoothed at 6 mm with a full width at half maximum Gaussian kernel. Each voxel timeseries was scaled to a mean of 100, and AFNI's 3dDeconvolve was then used to perform a voxel-wise regression analysis with six motion parameters regressed out. This analysis yielded a voxel-wise t-statistic which was then thresholded based on subsequent analysis as described below. EPI data sets were visually inspected for alignment with anatomical T1 datasets. For cases in which alignment was not acceptable, align_epi_anat.py was used to alight the anatomical T1 scan to the EPI data set in AFNI.

### Group-level activation analyses

EPI datasets were transformed to 3 × 3 × 3 mm resolution into Talairach space (Talairach and Tournoux, 1988), and AFNI's 3dttest++ was used to generate maps of average group-level activation for each time point. Group-level activation maps were clustered after using AFNI's 3dClustSim to calculate a minimum cluster size of 300 contiguous voxels needed for cluster-based correction for multiple comparisons (*p* ≤ 0.05).

For the subjects who had suffered a right-sided stroke with left-sided motor impairment, statistical maps of voxel-wise t-statistics were mirrored about the midline to produce scans demonstrating a stroke lesion in the left brain. These mirrored scans were then used in subsequent group analyses, such that as a group the stroke lesion is evidenced in the left hemisphere and the resulting motor impairment was in the right upper extremity. This process made the assumption that activity in the motor network is symmetric and that these mirrored scans would be otherwise comparable to scans from the subjects with left-sided strokes similar to other studies (Johansen-Berg et al., 2002a; Ward et al., 2003; Darling et al., 2007; Carter et al., 2010a; Confalonieri et al., 2012; Lotze et al., 2012; Stagg et al., 2012).

### LI calculations and analysis

Masks of significant clusters were made for each individual subject performing each task at each timepoint using these minimum clustersize values (range 163–310 contiguous voxels). These masks of significant clusters were created in subject space using the original subject-space voxel-wise maps of t-statistics and also in Talairach space using voxel-wise maps of t-statistics that had been transformed into Talairach space for each scan.

Three sets of ROI masks were created to examine the lateralization of motor function in these subjects to examine changes in laterality not only throughout the brain but also within smaller regions of the brain most relevant to motor function. The first was a pair of masks designed to capture activity throughout the whole brain (Whole Brain mask). This set was hand drawn in Talairach space to ensure complete coverage of the entire brain and then applied to the Talairached masks of significant clusters of activation for each subject. The second pair of masks (Motor Network mask) was constructed using spheres of radius 6 mm based on regions previously identified as parts of the motor network using an independent component analysis of whole-brain resting state fMRI scans (Shirer et al., 2012). The third pair of masks (Motor Cortex mask) consisted of only the cortical components from the Motor Network mask for each hemisphere. These latter two masks were resampled into subject space and then applied to the masks of significant clusters of activation for each subject. It is worth noting that because the motor network masks were originally based empirically on whole-brain resting state fMRI scans, these masks included areas of both primary and secondary areas of motor cortex and were not completely symmetric in shape. The cerebellum components of each half of the whole-brain and motor network masks were associated with cortical and subcortical structures on the opposite side of the brain because in a normal brain coordinated movement of a given hand is mediated by the contralateral brain hemisphere in conjunction with the ipsilateral cerebellum relative to the hand. A summary of the components of each mask is presented in Table S3.

LI was calculated for each fMRI scan using each of these three sets of masks at each of two thresholds to give a quantitative measure of finger tapping lateralization. The formula (V_I_−V_C_)/(V_I_+V_C_) was used to calculate each LI value, where V_I_ is the number of voxels in the ipsilesional hemisphere mask that show significant activation at a preset statistical threshold and V_C_ is the number of voxels in the contralesional hemisphere mask. Using this system, LI values above 0.2 are considered to represent ipsilesionally lateralized activity, those below −0.2 represent contralesionally lateralized activity, and those between −0.2 and 0.2 correspond to bilateral activity (Springer et al., 1999). Since LI values may change slightly when evaluated at different thresholds of significance (Pillai and Zaca, 2011), each mask was applied at the thresholds of *t* = 2.003 and *t* = 4 (which correspond to *p* < 0.05 and *p* < 0.0001 respectively) in order to evaluate potential trends at both a minimally stringent and a very stringent level of significance.

For subjects who had suffered a right-sided stroke with left-sided motor impairment, ROI masks were applied to individual masks of significant clusters without mirroring about the midline. LI analysis was performed using the formulas detailed above, with higher LI values corresponding to increasingly ipsilesional lateralization of finger tapping function. This process again made the assumption that activity in the motor network is symmetric.

A linear mixed-effects model was used to analyze raw LI values for each mask-threshold combination within data sets from the BCI therapy and control phases to assess for changes at each time point relative to baseline.

### LI-behavioral correlation analysis

At the individual level, values for change from baseline LI during finger tapping of the impaired hand were calculated at each time point. Values for change from baseline ARAT, baseline normalized SIS Hand Function domain, and baseline 9-HPT scores were also calculated at each time point for each subject able to complete each respective assessment. Data from subjects who exhibited floor or ceiling effects on a particular measure were not included in the LI-behavioral correlation analysis for that measure. Floor effects were noted when a subject was consistently unable to perform all tasks necessary to complete the assessment at all time points or when the minimum possible score was achieved for the assessment at each of the four time points within a given phase of the study. Ceiling effects were noted when a subject scored the maximum possible points at each of the four time points. Thus, instances of floor and ceiling effects captured zero functional change in the subject using a particular assessment over time. These changes in functional measures were then assessed for correlations with changes in LI using a Spearman's rank correlation analysis, making the assumption of independence among data points. However, because these data were collected using a repeated measures experimental design, some data points are not truly independent of one another. Therefore, relationships found to be statistically significant using the Spearman's rank correlation analysis were then re-analyzed using generalized estimating equations in order to further examine and validate the conclusions of the initial correlation model. Generalized estimating equations provide a method for examining the relationship between two variables that, unlike Spearman correlation analysis, does account for the repeated measures aspect of the data collected but also require the assumption that independent and dependent variables be named explicitly in the model design. All statistical analyses were performed in R statistical software version 3.0.1, which is freely downloadable at http://www.r-project.org/.

## Results

### Participant characteristics

At the time of this analysis, 11 subjects meeting inclusion and exclusion criteria had begun participation in this ongoing study. Among these participants, five were randomized to the BCI therapy group and had completed the BCI therapy phase, three had been assigned to the crossover control group and had completed both control and intervention phases, and data from three additional subjects that had been randomized to the crossover control group was available from the control phase of the study but not from the BCI therapy phase. Therefore, data used in this study represents information obtained from 11 individual subjects with control phase data available for six subjects and BCI therapy phase data available for eight subjects.

Participant characteristics are summarized in Table 1. Data from the experimental BCI therapy group comprised data from the BCI therapy phase of the experiment from subjects 1 to 8, while control group data comprised data from the control phase of the experiment from subjects 6 to 11. Among subjects who received BCI therapy, average age was 63 years (*SD* = 9.5 years), and average time from stroke onset was 13.13 months (*SD* = 8.44 months). More subjects had right-sided impairments than left-sided impairments, but this difference was not significant (*p* = 0.14). More subjects also had cortical strokes than non-cortical strokes, and more subjects were male than female; these differences were not significant (*p* = 0.36 for both comparisons).

**Table 1:** **Participant Characteristics**.

**Subject ID**	**Sex (M/F)**	**Age (years)**	**Handedness**	**Stroke location**	**Impaired hand**	**NIHSS (score)**	**Level of impairment**	**Time from stroke (months)**
1	M	52	R	L MCA	R	8	Severe	15
2	F	61	R	L frontal lobe	R	8	Severe	16
3	M	68	R	L centrum semiovale	R	0	Mild	3
4	M	66	R	L MCA	R	6	Severe	23
5	F	73	R	L MCA	R	0	Mild	2
6	F	75	R	R putamen	L	7	Severe	23
7	M	61	R	L basal ganglia	R	0	Mild	17
8	M	48	L	R pons	L	3	Moderate	6
9	M	56	R	L MCA	R	2	Moderate	11
10	M	48	R	R medulla	L	6	Severe	5
11	M	51	R	R MCA	L	4	Severe	16

*L, left; R, right; MCA, Middle cerebral artery*.

Among subjects from whom data was collected during from the control phase, average age was 56.5 years (*SD* = 10.34 years), and average time from stroke was 13 months (*SD* = 6.96 months). More subjects who completed the control phase of the experiment had left-sided impairments than right-sided impairments, and more had non-cortical stroke than cortical strokes, but these differences were not significant (*p* = 0.688 for both). Similar to subjects who received BCI therapy, more subjects were male than female, but this difference was not significant (*p* = 0.22). In both groups, more subjects were right-handed than left-handed, but this difference was again not significant (*p* = 0.14 for BCI therapy recipients; *p* = 0.22 among control phase subjects).

### Subject retention and compliance

Of the subjects enrolled, there were no drop outs due to subject desire to cease participation in the study or from any reported unpleasant experiences with the assessments or therapies administered. One participant, Subject 7, was not assessed or scanned at the final time point 1 month after the cessation of all BCI therapy due to scheduling conflicts between subject availability and scanner availability that prevented him from participating during the appropriate time window.

### Functional activation maps

**Figure 3:**
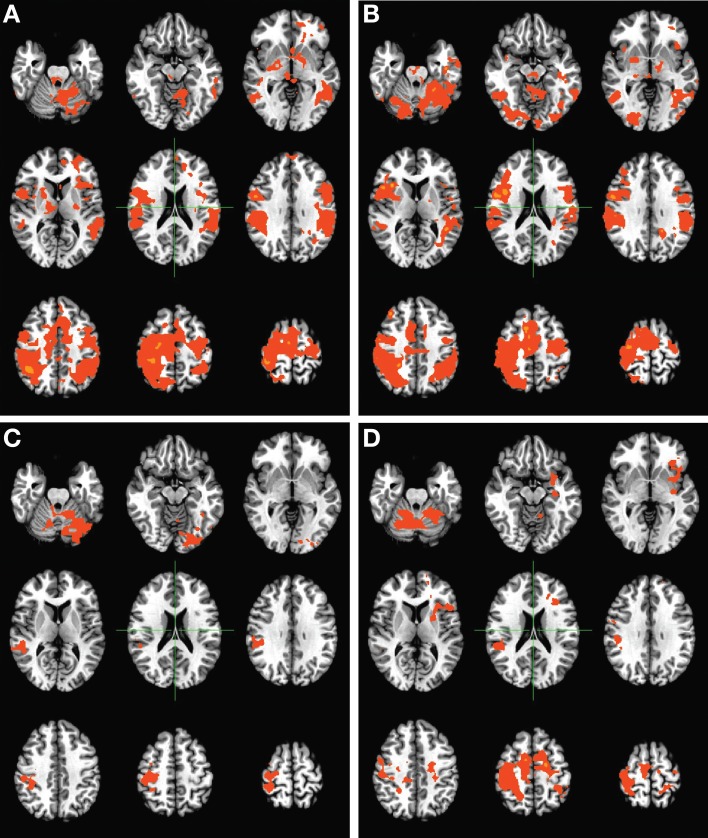
**Group level activation maps for finger tapping of the unimpaired hand during the BCI therapy phase of the experiment**. Panels demonstrate activation assessed **(A)** pre-therapy, **(B)** mid-therapy, **(C)** upon completion of therapy, and **(D)** 1 month after the cessation of all therapy, and show the development of a lateralized, focal pattern of activation with the administration of therapy. Maps are displayed according to radiological conventions, such that the right side of the image corresponds to the left hemisphere of the brain.

**Figure 4:**
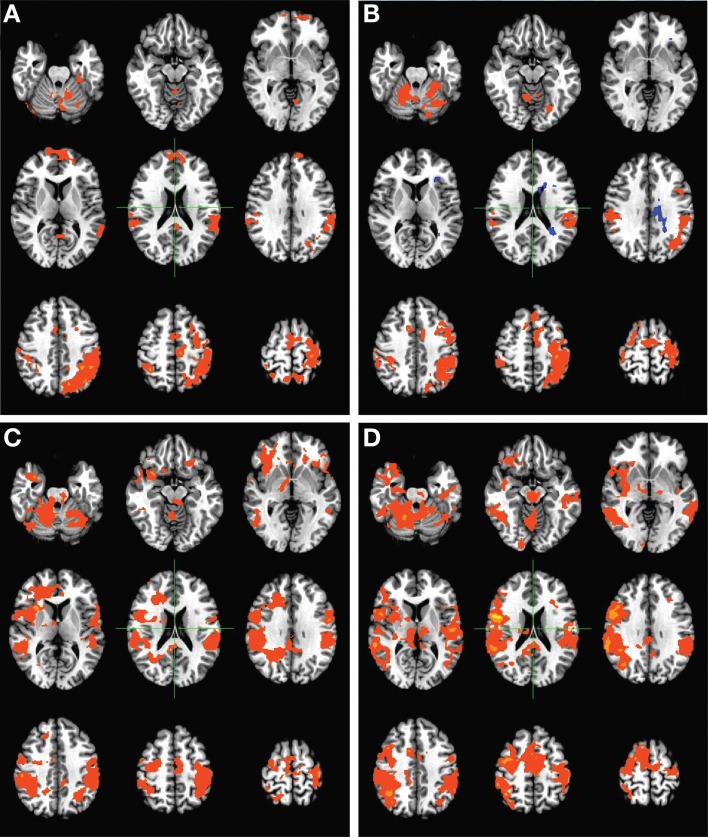
**Group level activation maps for finger tapping of the impaired hand during the BCI therapy phase of the experiment**. Panels demonstrate activations assessed **(A)** pre-therapy, **(B)** mid-therapy, **(C)** upon completion of therapy, and **(D)** 1 month after the cessation of all therapy, and show the development of a bilateral pattern of activation with increased recruitment of contralesional areas with the administration of therapy. Maps are displayed according to radiological conventions, such that the right side of the image corresponds to the left hemisphere of the brain.

Group-level activation maps during finger tapping of the unimpaired hand (Figure 3) showed changes in overall activation patterns, with baseline bilateral activation progressing to largely contralesionally lateralized activation over the course of therapy. These changes appear to have persisted to some degree 1 month after the cessation of BCI therapy. When considering group-level activation patterns observed during finger tapping of the impaired hand (Figure 4), a progression from more ipsilesional activity at baseline to bilateral activation was observed over the course of therapy. These changes again persisted 1 month after the cessation of BCI therapy. No such trends in group-level activation during finger tapping were noted over the course of the control phase of the experiment among subjects who completed the control phase of therapy.

### Group-level LI measures

**Figure 5:**
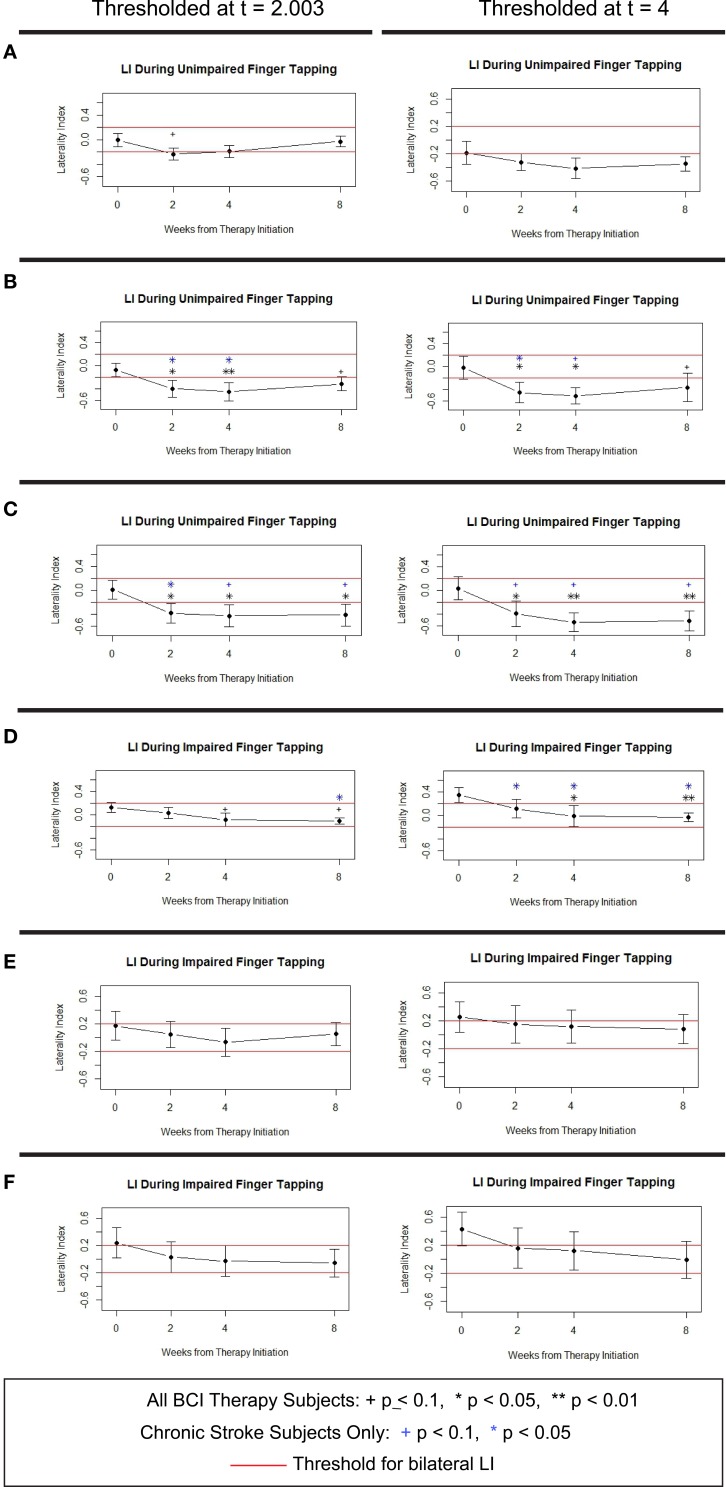
**Average LI values over time during unimpaired and impaired tapping from 8 subjects who received BCI therapy**. Values were calculated using **(A,D)** Whole Brain mask set, **(B,E)** Motor Network mask set, and **(C,F)** Motor Cortex mask set. Error bars shown are ±1 standard error of the mean. ^+^0.05 ≤ *p* < 0.1, ^*^p < 0.05, ^**^p < 0.01. Symbols in black show significant change from baseline for all 8 subjects who received BCI therapy. Symbols in blue indicate time points for significant change from baseline among the 6 subjects in the chronic stage of stroke who received BCI therapy. LI, Laterality Index.

Averaged LI values calculated using each set of ROI masks at each threshold during finger tapping of the unimpaired hand and during finger tapping of the impaired hand showed overall decreases in LI associated with administration of BCI therapy (Figure 5). This decrease resulted in an average shift from ipsilesionally lateralized activity at baseline to bilateral activity at all subsequent time points in 4 of 6 mask-threshold combinations during tapping of the impaired hand and a shift from average bilateral activity at baseline to contralaterally lateralized activity in all mask-threshold combinations mid-therapy that persisted after therapy in 5 of 6 mask-threshold combinations during tapping of the unimpaired hand. Analysis of these trends using linear mixed effects modeling revealed these LI decreases to be significantly different from baseline when using the Motor Network and Motor Cortex masks for LI calculations during unimpaired tapping and when using the Whole Brain mask to calculate LI during impaired tapping, as demonstrated by the black stars and plus symbols in Figure 5. These trends in LI changes with BCI therapy persisted even when only subjects in the chronic (at least 6 months from stroke onset) stage of stroke were analyzed (blue symbols in Figure 5). A similar analysis of LI values from the control phase of the experiment showed no similar trends and no significant changes from baseline at any time point for any of the six mask-threshold combinations.

### Functional assessment scores

Behavioral results for each subject at each time point are provided in the Tables S1, S2. Floor effects were observed during both the control (Subject 6 ARAT and 9-HPT, Subject 10 SIS Hand Function and 9-HPT, Subjects 8 and 11 9-HPT) and BCI therapy phases (Subject 1 SIS Hand Function and 9-HPT, Subject 2 all assessments, Subjects 4, 6, and 8 9-HPT) of the experiment. Subjects 1, 2, 4, 6, 8, 10, and 11 were unable to complete the 9-HPT task throughout the entire experiment and were therefore considered to be exhibiting a floor effect for this measure. These subjects were not included in subsequent analyses using data from the 9-HPT task. Ceiling effects were observed only during the BCI therapy phase of the experiment (Subject 3 ARAT). At the group level, the Sign test for paired samples was used to compare scores from each of the three behavioral measures mid-therapy, post-therapy, and 1 month after the end of therapy with baseline scores from the BCI therapy phase of the experiment and to compare scores from each assessment during the control phase with baseline control phase values. Changes in these scores were not significantly different from baseline at any time point, even when data from subjects exhibiting floor and ceiling effects were excluded from the comparison.

### LI-behavioral correlation analyses

A summary of the relationships observed between individual changes in LI and individual changes in behavioral measures during the BCI therapy phase of the experiment is provided in Table 2. Of the LI-behavioral relationships from the BCI therapy phase identified as significant at the *p* ≤ 0.05 level with the Spearman's approach, two remained significant when re-analyzed with GEE (*p* < 0.001 for both). Among LI-behavioral relationships examined from data obtained during the control phase of the experiment, only the relationship between changes in LI using the Whole Brain mask set at threshold *t* = 4 and changes in ARAT scores was found to be significant using Spearman's rank correlation analysis (*p* = 0.039), although this relationship did not survive when re-analyzed using GEE (*p* = 0.647).

**Table 2:** **Correlation analyses between LI changes and functional changes during the BCI therapy phase of the study**.

**Functional measure**	**Mask set**	**Threshold (t)**	**Spearman's rho**	***p*-value**
ARAT	Whole brain	2.003	−0.5751	^*^0.025
ARAT	Whole brain	4.000	−0.3773	0.166
ARAT	Motor network	2.003	−0.315	0.253
ARAT	Motor network	4.000	−0.3556	0.193
ARAT	Motor cortex	2.003	−0.4945	^+^0.061
ARAT	Motor cortex	4.000	−0.6031	^*^0.017
SISHF	Whole brain	2.003	−0.3203	0.226
SISHF	Whole brain	4.000	−0.5634	^#^^*^0.023
SISHF	Motor network	2.003	−0.2734	0.305
SISHF	Motor network	4.000	−0.3967	0.143
SISHF	Motor cortex	2.003	−0.2493	0.352
SISHF	Motor cortex	4.000	−0.3333	0.225
9-HPT	Whole brain	2.003	0.4444	0.171
9-HPT	Whole brain	4.000	0.3241	0.331
9-HPT	Motor network	2.003	0.2315	0.493
9-HPT	Motor network	4.000	0.205	0.57
9-HPT	Motor cortex	2.003	0.6296	^#^^*^0.038
9-HPT	Motor cortex	4.000	0.1429	0.694

* Significant at p < 0.05;

+ trend toward significance at 0.05 < p < 0.1;

# *also significant with generalized estimating equations approach*.

**Figure 6:**
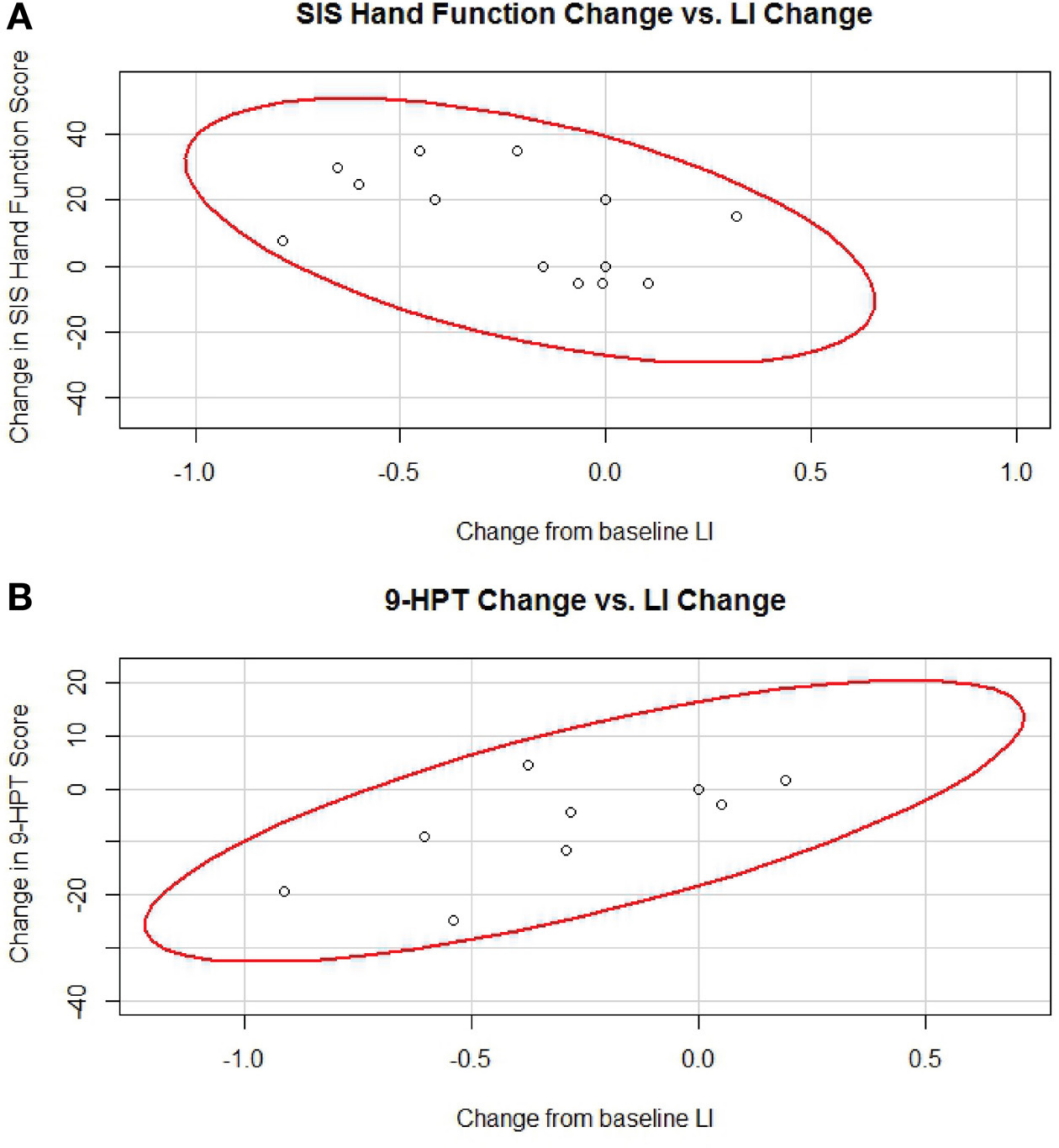
**Significant correlations between changes in LI and changes in behavioral measures. (A)** Relationship between individual changes in LI and individual changes in SIS Hand Function. Data from 4 subjects representing 15 data points assessed. **(B)** Relationship between individual changes in LI and individual changes in 9-HPT. Data from 3 subjects representing 11 data points assessed. Red lines represent data ellipses at the 95% confidence level. LI, Laterality Index; SIS, Stroke Impact Scale; 9-HPT, Nine-Hole Peg Test.

The correlations found to be significant using Spearman's rank correlation analysis that remained significant upon secondary analysis using generalized estimating equations are shown in Figure 6.

It is also notable that the calculated estimates for Spearman's rho (Table 2) are consistent in sign among correlation analyses performed for the same functional measure (i.e., changes in ARAT and SIS Hand Function scores were all estimated to be negatively correlated with changes in LI, while changes in 9-HPT times were all estimated to be positively correlated with changes in LI). Furthermore, while the direction of the correlations is switched for the 9-HPT measure relative to the other two measures, it is important to interpret this difference while keeping in mind the scoring methods for each. In particular, ARAT and SIS Hand Function are scored positively, with improvements in performance reflected in higher scores, while the 9-HPT scores are reported as the time needed to complete the task in seconds, with improvements in (i.e., faster) performance reflected in lower times. Thus, all estimated Spearman correlations using data from the BCI therapy phase of the experiment were actually consistent with an association between decreases in LI and gains in behavioral measures. In contrast, this consistency was not noted in correlation analyses examining potential relationships between changes in LI values and changes in functional measures assessed during the control phase of the experiment.

## Discussion

The findings in this preliminary report on the neuroplastic effects of BCI therapy when applied to stroke rehabilitation show changes in task-related brain activation induced by interventional rehabilitative therapy using a closed-loop neurofeedback BCI device. Specifically, group-level changes in the patterns of brain activation associated with finger tapping of each hand were observed with the administration of BCI therapy, with tapping of the impaired hand producing a more bilateral activity pattern post-therapy and with tapping of the unimpaired hand eliciting more lateralized activity involving the hemisphere contralateral to the unimpaired hand (Figures 3–4).

These patterns of change noted in our activation maps were further quantified using LI analyses, which demonstrated shifts in activity with BCI therapy from the ipsilesional hemisphere to greater involvement of the contralesional hemisphere (i.e., to bilateral activity) during tapping of the impaired hand and increased lateralization of activity to the hemisphere contralateral to the unaffected hand (i.e., the contralesional hemisphere) during tapping of the unimpaired hand. These LI changes were observed using multiple sets of brain masks at multiple thresholds and generally persisted even when the two subacute stroke subjects in the sample were removed from the analysis (Figure 5). The persistence of these findings among chronic stroke subjects argues against this effect arising from a spontaneous recovery process, as chronic stroke is considered to be a time when little or no spontaneous recovery is expected (Nakayama et al., 1994). Furthermore, no such trends or changes in LI were observed in a similar series of scans obtained during a control period in which no BCI therapy was given. That the subanalysis of chronic stroke patients showed LI changes with BCI therapy further supports the hypothesis that these changes were effected, at least in part, by the BCI therapy in that the absence of such effects in the control data set cannot be attributed to the fact that two fewer subjects were assessed during the control phase compared to the full group who received BCI therapy (*n* = 8) as the two groups (chronic stroke patients who received BCI therapy and subjects who completed the control phase of the experiment) were of equal size (*n* = 6). Therefore, if the lack of results in the control group were due merely to a power issue, no such results should be present in the subanalysis using only the six chronic stroke subjects who received BCI therapy. These preliminary differences suggest that such changes in LI may be induced by administration of the BCI therapy used in this study. The persistence of these LI changes up to 1 month after the conclusion of BCI therapy further suggests the possibility for lasting effects to be achieved with the use of this rehabilitative approach.

Taken together, the changes observed in brain activation patterns and LI values during tapping of the impaired and unimpaired hands associated with the administration of BCI therapy suggest that there may exist differential patterns of response to the same treatment with interventional BCI therapy between the impaired and unimpaired sides of the motor system. These differential responses may be characterized both in the direction of LI change (increasingly lateralized in the case of unimpaired movement vs. increasingly bilateral in the case of impaired movement) as well as in the time course or dose-response of each, with more time or therapy needed before a significant reorganization effect can be observed in LI during an impaired task relative to that needed to observe a significant reduction in LI during an unimpaired task. This difference in brain organization after stroke and re-organization in response to BCI therapy between the ipsilesional and contralesional hemispheres highlights the importance of examining neural responses to treatments such as BCI therapy in stroke survivors even after these systems have been validated in other populations, as it calls into question the degree to which patterns of brain reorganization demonstrated in early studies using healthy normal subjects or limited to stroke subjects with intact cortical functioning (Caria et al., 2011; Ramos-Murguialday et al., 2013) can be generalized to stroke patients and other populations with significant cortical pathologies.

Changes in LI observed during and after the administration of BCI therapy also correlated to changes in behavior observed during the same time period when using some of the mask-threshold combinations. Of the four correlations found to be significant using Spearman's rank-correlation analysis on this data from the BCI therapy phase of the experiment, two remained significant when reanalyzed using a generalized estimating equations approach. The relationship suggested by both significant and non-significant estimated Spearman's coefficients was also consistent across all mask-threshold combinations, with more bilateral LI values correlating to greater functional gains during tapping of the impaired hand across all three behavioral assessments. In contrast, similar correlation analyses performed on data from the control phase of the experiment found only one significant correlation using Spearman's rank-correlation analysis which did not survive when reanalyzed using generalized estimating equations, and the same consistency among the direction of LI change association with improvements in behavioral measures was not observed. The finding of only some mask-threshold combinations and not others giving rise to significant relationships with the behavioral assessments may be due to limitations of the small sample size used, rendering the current preliminary analysis underpowered to detect more subtle relationships between LI changes and functional gains. It is also possible that some mask/threshold combinations are better suited to capturing brain-behavior relationships depending on the behavioral assessment used.

While overall changes in behavioral outcomes during the period of BCI therapy administration were not found to be statistically significant at the group level, the correlations revealed by analyses of individual LI and functional changes suggest the presence of or potential for some degree of sub-clinical functional improvement not well-captured by the assessments used. Indeed, the presence of such correlations between gains in 9-HPT performance and LI changes with BCI therapy further demonstrates the importance of capturing small subclinical gains in function. As only the three least impaired subjects in the group studied who received BCI therapy were able to complete this assessment, changes in 9-HPT performance and LI may show neurological changes that correlate with functional improvement during and after BCI therapy administration and allow us to begin characterizing this relationship present even in highly functioning stroke survivors. It may also be worth noting that while the ARAT and 9-HPT are objective measures of motor function, scores for SIS Hand Function constitute a more subjective measure reliant on self-report. Thus, these changes in brain activity being detected by fMRI may be effecting change both in a subjective measure of how well the subjects believe they are performing, as well as in objective measures of the amount of ability that they are able to demonstrate using standardized motor function tests.

When considering previous work examining correlations between changes in LI and behavioral measures after BCI therapy, one study by Ramos-Murguialday and colleagues found that BCI therapy induced changes in LI of activity in the motor and premotor cortices during attempted movement of the paretic hand, shifting LI toward the ipsilesional hemisphere and correlating the magnitude of these individual shifts with post-therapy motor performance. A similar finding was documented in a case study of a thalamic stroke survivor, in which treatment with BCI therapy was associated with both functional gains and a shift from bilateral brain activation to lateralized activation in the ipsilesional hemisphere (Caria et al., 2011). While the pattern of functional brain reorganization with BCI therapy for stroke rehabilitation documented in these studies appears to describe an LI-behavior correlation opposite that in our findings, it is important to note that these findings by Ramos-Murguialday and Caria were specific to subjects who had suffered subcortical strokes. Thus, these associations may not be generalizable to patients who have suffered cortical damage, as was true of the majority of subjects in the present study. In fact, the pattern of increasingly lateralized LI induced by BCI therapy observed by the Ramos-Murguialday study is similar in pattern and direction to that observed in the unlesioned hemisphere of our subjects during tapping of the unimpaired hand. Our study builds on findings from a feasibility study which indicated that the activity in the brain hemisphere ipsilateral to the affected hand (i.e., the contralesional hemisphere) can be used as control signals for a BCI system designed to respond to movement intention (Bundy et al., 2012) along with the knowledge that in some patients infarction and hypoperfusion may shift areas of activation toward the intact hemisphere in other domains such as language (Prabhakaran et al., 2007). Together, these findings suggest that increased laterality toward the contralateral hemisphere during attempted hand movement may be a typical pattern of response to this type of BCI training in unlesioned cortex.

In designing interventional BCI systems for rehabilitation therapy, such as that used in the present study and in studies referenced previously, there remains a fair amount of uncertainty regarding what patterns of brain reorganization after stroke are optimal in allowing for rehabilitation after stroke and what differences there may be in such patterns among various subpopulations of stroke survivors. Two of the most prevalent competing theories take different stances regarding whether recruitment or activation of motor areas in the contralesional hemisphere during the chronic stage of stroke is beneficial or detrimental to functional recovery. Each of these views has influenced the development of therapeutic approaches intended to facilitate better rehabilitative outcomes for patients in the subacute and chronic stages of stroke who have reached a functional plateau with traditional standard therapies alone.

Some of these new approaches promote the activation of ipsi- or peri-lesional cortex with or without additionally discouraging activation of contralesional motor areas, effectively encouraging the re-lateralization of functional motor activity to the lesioned hemisphere. These methods stem from observations in studies of spontaneous recovery suggesting that the best recovery outcomes are accompanied by an eventual return to pre-stroke lateralization for these functions. This pattern has been documented in both motor (Turton et al., 1996; Traversa et al., 1997, 1998; Marshall et al., 2000; Calautti et al., 2001; Johansen-Berg et al., 2002b; Richards et al., 2008; Dimyan and Cohen, 2011) and language (Saur et al., 2006; Saur and Hartwigsen, 2012) domains in chronic stroke patients. Additional evidence to support this approach comes from studies of functionally associated neuroplastic change to targeted rehabilitation interventions, which have been shown to correlate with neural plastic changes in the lesioned hemisphere. A systematic review and meta-analysis by Richards and colleagues in 2008 of movement-dependent approaches in stroke recovery including CIMT, task practice, virtual reality training, and bilateral movements also supported this model of ipsilesional lateralization coinciding with better recovery in the sub-acute and chronic phases of stroke (Richards et al., 2008).

In contrast, other approaches have been developed that aim to promote the development of new or secondary pathways in the functional organization of the brain, recruiting areas of the contralesional brain to assist in the motor control of the impaired hand. This theory is based on the fact that 8–10% of corticospinal tract fibers project ipsilaterally in humans and other primates (Galea and Darian-Smith, 1994; Hoyer and Celnik, 2011) with the hypothesis that in some stroke patients, the lesioned hemisphere ceases to inhibit these ipsilateral motor projections which can then be used for control of the impaired arm (Stoeckel and Binkofski, 2010). There is some empirical evidence strengthening the case for attempting to maximize ipsilateral control of a paretic limb, with stroke survivors showing reductions in recovered motor performance after TMS of the contralesional hemisphere (Lotze et al., 2006) and one small study of showing contralesional activation increased with CIMT (Kopp et al., 1999). Motor performance has also been found to correlate more strongly with interhemispheric than ipsilesional functional connectivity following stroke (Carter et al., 2010a). Such studies support the idea that stroke recovery relies at least in part on the functional coordination and activation of the contralesional brain. It has been shown that motor performance in the chronic stage of stroke can be related in particular to the degree of corticospinal tract damage sustained by stroke patients (Stinear et al., 2007), suggesting that this tract plays an integral part in motor recovery. For those who are severely impaired due to a greater burden of irreversible corticospinal tract damage, the recruitment of additional cortical areas may be necessary (Newton et al., 2006; Jayaram and Stinear, 2008), including alternate contralesional pathways, for maximal recovery of motor control. Even with the inclusion of mildly impaired stroke patients, the results of the current study appear to support the participation of the unlesioned hemisphere in the neural reorganization after stroke, which may facilitate recovery of function.

It has been shown that cortical activity in both humans and non-human primates reliably encodes a representation of ipsilateral arm movement (Ganguly et al., 2009) and that differential cortical patterns can be reliably detected within a single hemisphere to differentiate movements of the ipsilateral and contralateral hands (Wisneski et al., 2008). Building on this work, new BCI devices have been developed that can be controlled using only ipsilateral neural signals (Bundy et al., 2012). These devices are being studied primarily in severely impaired subjects most likely to benefit from therapies using this type of approach, as individuals in this stroke population are those most likely to experience a motor recovery process that relies more heavily on new or accessory pathways after significant damage to the ipsilesional corticospinal tract (Bundy et al., 2012). It is important to note that the targeting of these new BCI devices promoting ipsilateral control of the impaired arm to severely impaired stroke survivors, while theoretically motivated, does not necessarily mean that others with less severe impairments could not also benefit from such therapies. Currently, there remains insufficient evidence to determine definitively which of these newer therapies are best suited to stroke survivors with different levels of impairment.

It is also important to remember that the conclusions regarding optimal patterns of functional brain reorganization from the study of one therapy modality may not be generalizable to other therapy modalities such as BCI. For example, two studies following similar populations of chronic stroke patients (all but one with subcortical lesions) found motor function gains to be associated with increased ipsilesional activation laterality or with increased contralesional recruitment during motor tasks of the affected hand after using BCI and gesture therapy respectively (Orihuela-Espina et al., 2013; Ramos-Murguialday et al., 2013). With this in mind, it may not be necessary to identify an optimal pattern of change common across therapy modalities as long as respective approaches are able to induce neuroplastic change and maximize functional recovery in chronic stroke patients. Understanding these patterns of neuroplastic change might then serve simply to allow for optimization within the application of a particular modality.

Although our study had a small and somewhat heterogenous sample of stroke survivors both in terms of motor impairment (mild to severe) and chronicity of stroke (subacute to chronic), BCI induced consistent brain changes along with significant brain-behavioral associative changes in these patients over time that were not observed during a control period in which no BCI therapy was administered. Changes in neural activation patterns were observed with BCI therapy in both primary and secondary motor areas in this population of stroke patients, and these activation patterns were then quantified in LI calculations using masks that included regions of both primary and secondary motor areas. It is promising that these findings persisted across multiple mask sets at multiple thresholds, some of which were found to have significant correlations to changes in behavioral measures when using both correlation and GEE models. Although the findings in this study are based on a small group of subjects, a subanalysis of only the chronic stroke patients who received BCI therapy showed consistent effects, supporting the hypothesis that administration of this BCI therapy helped to produce the effects observed. The lack of changes in the control group also argues against attributing the effects observed to spontaneous recovery, as the control group was similar to the group that received BCI therapy in numerous measures such as age, sex, handedness, and chronicity of stroke (Table 1).

Even though these preliminary analyses were potentially underpowered for the detection of smaller changes in LI earlier in the therapy time course and for the more consistent detection of significant relationships between LI change and behavioral gains, future studies incorporating a larger sample of stroke patients will be able to better characterize these patterns of LI change with BCI therapy and to detect more subtle relationships that these LI changes may have with concurrent behavioral gains. Larger sample sizes will also allow for the investigation of potential cross over effects into domains not directly targeted by the BCI device, such as strength, participation, and activities of daily living, as such effects are expected to be more subtle than those observed in the targeted domain of upper extremity motor function. The use of additional assessments such as the Fugl–Meyer and Chedoke Arm and Hand Inventory may also allow for the detection of more subtle changes in functional capability that may accompany this type of therapy such that floor effects and ceiling effects might be a smaller limitation of future studies. Similarly, future studies on the effects of BCI therapy for stroke rehabilitation will be needed not only to characterize more subtle patterns of neuroplastic change that may result from this type of treatment but also to determine the optimal mask and threshold parameters that might allow LI to be used as an imaging biomarker to assess and track recovery in individuals receiving this type of therapy.

The findings of this study may provide further insight into what theoretical approaches might be optimal in designing future neurofeedback devices and paradigms for motor recovery after stroke, particularly with regard to which patterns of brain activity might be actively rewarded or discouraged by future systems in order to elicit the greatest possible recovery in these patients. In determining whether increased ipsilesional lateralization vs. increased bilateral recruitment is optimal for motor recovery, a review by Dimyan and Cohen documented the former pattern as associated with spontaneous recovery while a small number of interventional and disruptive studies supported the latter in the development of novel interventions to promote neural reorganization in poststroke rehabilitation (Dimyan and Cohen, 2011). This is consistent with the possibility that there exist multiple patterns of neural reorganization that facilitate better recovery after stroke and that such patterns may be modulated with the administration of interventional therapy using newer technologies that are different from those evidenced during spontaneous recovery.

In exploring optimal device design, it may be more important with developing BCI therapies than with the development of other therapy modalities to determine what pattern of neural activity corresponds to the greatest behavioral gains because the BCI system can be programmed to preferentially reward predefined neural signatures. If such patterns for maximal rehabilitation are discovered, it is not difficult to envision the development and clinical use of customized BCI therapy interventions based on the functional and neurological profile of an individual patient, a form of what have been previously termed as “prescriptive” interventions (Carter et al., 2010b).

As the field of interventional rehabilitative therapy using BCI continues to develop, there remains a need for larger studies incorporating an element of randomized control to better understand and characterize the effects being elicited by the BCI therapy itself and also to identify subpopulations of stroke survivors with differential responses to this type of therapy. Ideally, such studies will also incorporate a wide range of behavioral assessments, neuroimaging measures, and connectivity analyses to capture and provide a comprehensive understanding of the various brain and behavior changes induced by BCI therapy and the relations between them.

## Author contributions

Brittany M. Young assisted in subject recruitment, data collection, data analysis, and writing. Zack Nigogosyan assisted with data collection, data analysis, and writing. Léo M. Walton assisted with data collection and writing. Jie Song assisted with subject recruitment and data collection. Veena A. Nair assisted with subject recruitment, data collection, data analysis, and writing. Scott W. Grogan assisted with data collection. Mitchell E. Tyler provided TDU hardware and expertise. Dorothy F. Edwards assisted with study design and outcome measure selection and interpretation. Kristin Caldera assisted with subject referral and recruitment. Justin A. Sattin assisted with study design and subject recruitment. Justin C. Williams is one of two lead PI's on this project and supervised the technical and engineering aspects of the work. Vivek Prabhakaran is one of two lead PI's on this project and supervised the neuroimaging aspects of this work.

### Conflict of interest statement

A pending patent on the closed-loop neurofeedback device used for the therapy administered in this study (Pending U.S. Patent Application No. 12/715,090) was filed jointly by the two lead investigators Justin C. Williams and Vivek Prabhakaran. Otherwise, the authors declare that the research was conducted in the absence of any commercial or financial relationships that could be construed as a potential conflict of interest.

## Supplementary material

Table S1 **Functional outcomes as assessed by SIS Hand Function, Action Research Arm Test, and Nine-Hole Peg Test during the BCI Therapy phase of the experiment**. Subjects who were unsuccessful at completing the 9-HPT within 5 min were noted as unable to perform this assessment. Subject numbers in this table match those in Table 1, which provides participant characteristics. SIS, Stroke Impact Scale; ARAT, Action Research Arm Test; 9-HPT, Nine-Hole Peg Test.

Table S2 **Functional outcomes as assessed by SIS Hand Function, Action Research Arm Test, and Nine-Hole Peg Test among Subjects completing assessments during the control phase of the experiment**. Subjects who were unsuccessful at completing the 9-HPT within 5 min were noted as unable to perform this assessment. Subject numbers in this table match those in Table 1, which provides participant characteristics. SIS, Stroke Impact Scale; ARAT, Action Research Arm Test; 9-HPT, Nine-Hole Peg Test.

Table S3 **Components of masks used for LI calculations**. Mask regions with “motor and premotor cortex” included components of the precentral, postcentral, middle frontal, medial frontal, and superior frontal gyri. LI, Laterality Index.
