# Supplementary material for: Changes in functional brain organization and behavioral correlations after rehabilitative therapy using a brain-computer interface
Source: Front Neuroeng. 2014 Jul 15;7:26. doi: 10.3389/fneng.2014.00026 (PMC4097124; doi:10.3389/fneng.2014.00026)
Supplement: Table S1 — Functional outcomes as assessed by SIS Hand Function, Action Research Arm Test, and Nine-Hole Peg Test during the BCI Therapy phase of the experiment. Subjects who were unsuccessful at completing the 9-HPT within 5 min were noted as unable to perform this assessment. Subject numbers in this table match those in Table 1, which provides participant characteristics. SIS, Stroke Impact Scale; ARAT, Action Research Arm Test; 9-HPT, Nine-Hole Peg Test. [file DataSheet2.DOCX]

**Table S1.** Functional outcomes as assessed by SIS Hand Function, Action Research Arm Test, and Nine-Hole Peg Test during the BCI Therapy phase of the experiment. Subjects who were unsuccessful at completing the 9-HPT within five minutes were noted as unable to perform this assessment. Subject numbers in this table match those in Table 1, which provides participant characteristics. SIS = Stroke Impact Scale. ARAT = Action Research Arm Test. 9-HPT = Nine-Hole Peg Test.

| Subject | Timepoint | SIS Hand Function | ARAT | 9-HPT (seconds) |
| --- | --- | --- | --- | --- |
|  |  | **Possible Score Range** | | |
|  |  | **0-100** | **0-57** | **0-300** |
| 1 | Pre-therapy | 0 | 0 | Not Able |
|  | Mid-therapy | 0 | 3 | Not Able |
|  | Post-therapy | 0 | 0 | Not Able |
|  | 1 Month Post-therapy | 0 | 0 | Not Able |
| 2 | Pre-therapy | 0 | 0 | Not Able |
|  | Mid-therapy | 0 | 0 | Not Able |
|  | Post-therapy | 0 | 0 | Not Able |
|  | 1 Month Post-therapy | 0 | 0 | Not Able |
| 3 | Pre-therapy | 40 | 57 | 66 |
|  | Mid-therapy | 55 | 57 | 55.5 |
|  | Post-therapy | 70 | 57 | 46.5 |
|  | 1 Month Post-therapy | 75 | 57 | 41.25 |
| 4 | Pre-therapy | 0 | 3 | Not Able |
|  | Mid-therapy | 0 | 0 | Not Able |
|  | Post-therapy | 0 | 0 | Not Able |
|  | 1 Month Post-therapy | 0 | 0 | Not Able |
| 5 | Pre-therapy | 50 | 56 | 38.36 |
|  | Mid-therapy | 70 | 46 | 35.355 |
|  | Post-therapy | 50 | 54 | 40 |
|  | 1 Month Post-therapy | 57.5 | 57 | 29.45 |
| 6 | Pre-therapy | 5 | 0 | Not Able |
|  | Mid-therapy | 0 | 0 | Not Able |
|  | Post-therapy | 0 | 0 | Not Able |
|  | 1 Month Post-therapy | 0 | 0 | Not Able |
| 7 | Pre-therapy | 75 | 53 | 66.485 |
|  | Mid-therapy | 75 | 57 | 71 |
|  | Post-therapy | 75 | 54 | 55 |
| 8 | Pre-therapy | 10 | 27 | Not Able |
|  | Mid-therapy | 30 | 28 | Not Able |
|  | Post-therapy | 35 | 40 | Not Able |
|  | 1 Month Post-therapy | 45 | 43 | Not Able |

**Table S2.** Functional outcomes as assessed by SIS Hand Function, Action Research Arm Test, and Nine-Hole Peg Test among Subjects completing assessments during the control phase of the experiment. Subjects who were unsuccessful at completing the 9-HPT within five minutes were noted as unable to perform this assessment. Subject numbers in this table match those in Table 1, which provides participant characteristics. SIS = Stroke Impact Scale. ARAT = Action Research Arm Test. 9-HPT = Nine-Hole Peg Test.

| Subject | Timepoint | SIS Hand Function | ARAT | 9-HPT (seconds) |
| --- | --- | --- | --- | --- |
|  |  | **Possible Score Range** | | |
|  |  | **0-100** | **0-57** | **0-300** |
| 6 | Baseline | 0 | 0 | Not Able |
|  | 2 Weeks | 0 | 0 | Not Able |
|  | 4 Weeks | 0 | 0 | Not Able |
|  | 8 Weeks | 5 | 0 | Not Able |
| 7 | Baseline | 55 | 54 | 106 |
|  | 2 Weeks | 35 | 57 | 76.5 |
|  | 4 Weeks | 50 | 51 | 74.5 |
|  | 8 Weeks | 75 | 53 | 66.5 |
| 8 | Baseline | 10 | 26 | Not Able |
|  | 2 Weeks | 0 | 27 | Not Able |
|  | 4 Weeks | 20 | 32 | Not Able |
|  | 8 Weeks | 10 | 28 | Not Able |
| 9 | Baseline | 30 | 54 | 67 |
|  | 2 Weeks | 45 | 57 | 41.2 |
|  | 4 Weeks | 45 | 57 | 42 |
|  | 8 Weeks | 55 | 54 | 39.4 |
| 10 | Baseline | 0 | 0 | Not Able |
|  | 2 Weeks | 0 | 0 | Not Able |
|  | 4 Weeks | 0 | 0 | Not Able |
|  | 8 Weeks | 0 | 3 | Not Able |
| 11 | Baseline | 25 | 2 | Not Able |
|  | 2 Weeks | 0 | 9 | Not Able |
|  | 4 Weeks | 35 | 3 | Not Able |
|  | 8 Weeks | 20 | 3 | Not Able |

**Table S3.** Components of masks used for LI calculations. Mask regions with “motor and premotor cortex” included components of the precentral, postcentral, middle frontal, medial frontal, and superior frontal gyri. LI = Laterality Index.

| ROI Mask Set | ROI Mask Name | Mask Components |
| --- | --- | --- |
| Whole Brain | Left Brain | Left cortex  Left subcortical structures Right cerebellum |
|  | Right Brain | Right cortex  Right subcortical structures  Left cerebellum |
| Motor Network | Left Motor Network | Left motor cortex  Left thalamus  Right cerebellum |
|  | Right Motor Network | Right motor cortex  Right thalamus  Left cerebellum |
| Motor Cortex | Left Motor Cortex | Left motor cortex |
|  | Right Motor Cortex | Right motor cortex |
