# Supplementary material for: Changes in functional brain organization and behavioral correlations after rehabilitative therapy using a brain-computer interface
Source: Front Neuroeng. 2014 Jul 15;7:26. doi: 10.3389/fneng.2014.00026 (PMC4097124; doi:10.3389/fneng.2014.00026)
Supplement: Supplementary file 2 [file DataSheet1.DOCX]

**Supplementary Materials and Methods**

*Subject retention and compliance*

Of the subjects enrolled, there were no drop outs due to subject desire to cease participation in the study. One participant, Subject 7, was not assessed or scanned at final time point one month after the cessation of all testing due to scheduling conflict that prevented him from participating during the appropriate time window. Due to technical issues, not all subjects completed all ten runs of each therapy session stage at all therapy sessions.

*fMRI data quality assurance*

Reconstruction of all scan sessions from all subjects was successful such that no scans needed to be excluded due to factors such as excessive motion. However, not all subjects completed both left hand and right hand finger tapping tasks at each time point. Of note, Subject 1 did not perform finger tapping of the impaired right hand at his pre-therapy assessment, Subject 2 did not complete either of the functional finger tapping scans at her post-therapy assessment, and Subject 7 was not assessed one month after the cessation of therapy due to scheduling issues (see *Subject Retention and Compliance*)

*Sequence of interventional therapy sessions*

Each session of interventional therapy using the brain-computer interface device consisted of four stages. Subjects began each session in stage one, performing attempted and imagined movement of each hand alternating with periods of rest. Each of these conditions (i.e. attempted movement of the right (or left) hand, imagined movement of the right (or left) hand, and rest) are prompted at least ten times, with a duration of four seconds per prompt. These initial trials are open-loop tasks in which no performance feedback is given. Data from these open-loop trials is analyzed offline to determine the appropriate EEG-based control features for the subsequent closed-loop tasks. This initial calibration task and its application to control feedback during later stages of the therapy session is based on previously described processes (*70*). Selection of these control signals was not necessarily limited to regions of the primary motor cortex, as it has been shown that control of a BCI cursor in similar motor task does not need to be trained on motor cortex areas but can also use additional areas of the brain such as the sensory cortex (*71, 72*), which is particularly useful for subjects with extensive motor cortex damage

The next stage of the intervention involves a closed-loop condition in which the subject is presented with real time visual feedback which allows them to learn how to modulate their cortical activity. Visual feedback is presented in the context of a game in which the subject is presented with a target on the left or right side of the screen and instructed to move a cursor from the center of the screen to the target by using attempted hand movement. The feedback component of this visual display is the lateral movement of the cursor toward or away from the on-screen target, which is controlled in real time by the subject’s EEG signals. Subjects perform attempted hand movements during all trials in this stage as well as in all trials of all subsequent stages. Cortical activity related to attempted movement of the right (left) hand as detected by EEG is translated into rightward (leftward) movement of the on-screen cursor. If the subject is able to maneuver the cursor to the target, the trial is considered a success. Trials are considered unsuccessful if the cursor fails to reach the target after five seconds, at which point the trial is aborted. Each run consists of ten trials, with each trial randomly presenting one of four possible targets to the subject. A goal of at least ten runs is presented to the subject during this stage and all subsequent stages. Subsequent stages also use the same game play paradigm with the incorporation of additional forms of feedback.

The third stage of the intervention session is similar to the second with the addition of FES to muscles of the impaired arm to assist with the impaired attempted movement. FES is triggered when cortical activity related to attempted movement of the impaired limb is detected by EEG and the subject has been cued to attempt movement of the impaired hand (i.e. when the target is on the same side as the impaired limb). Thus, since both cursor movement and FES are controlled by the same set of EEG signals, FES is only applied when the cursor moves correctly toward the target on the impaired side of the body. This triggering of the FES is significant in that it ensures that only consistent, desired patterns of brain activity associated with attempted movement of the impaired hand are rewarded with feedback from the FES device.

The fourth and final stage of the intervention session is again similar to the second and third stages with TS feedback administered along with visual and FES feedback. TS feedback parallels visual feedback in that it provides continuous electrotactile stimulation of the tongue during each trial. Furthermore, TS has been shown to provide sufficient feedback to enable a subject to use BCI devices with TS alone in the absence of visual or other tactile feedback (*73*) and has also been implicated in priming neuromodulation (*74*). TS with this device is organized in a grid that delivers electrical stimulus representing the positions of the on-screen cursor and target onto the tongue.

For subjects achieving good control of the stimuli who expressed the desire for a more challenging task, the game was made more difficult by varying game play parameters (e.g. changing the sizes of the targets or cursors) to keep the subjects interested. The advancement of game play parameters to produce a more difficult task typically occurred only after the first week of training had been completed. Subjects were allowed to take short rest breaks between stages if desired.

*Statistical analyses*

It is also important to acknowledge the limitations of the statistical analyses used in this study. In particular, the correlation analysis used to initially examine the correlations between changes in LI and changes in behavioral measures relies on the mathematical assumptions that the variables have a bivariate normal distribution, and that the observed data points are independent. However, it is clear from the study design that at least one of these assumptions, particularly the assumption of independence among data points, does not hold as the study used a repeated measures design. Performing a correlation analysis without fulfilling this assumption can result in an underestimation of the standard error and p-value calculated for the correlation between the variables examined. Therefore, the use of the generalized estimating equation (GEE) analysis as a secondary analysis helps to address the limitations of the initial correlation approach. Generalized estimating equations do not require the assumptions of independence among data points and can be used to analyze data from repeated measures studies and are generally more powerful in their ability to tease apart more complicated relationships that may exist. However, these analyses may require larger data sets in order to best elucidate such subtle relationships. Therefore, given the limited amount of data available from the size of the current cohort, in this study we began by making the assumptions needed for a Pearson’s r correlation analysis and then used GEE as a secondary analysis to support the findings of this initial analysis. The concordance of significant findings between the correlation analysis and GEE models suggests that a relationship does exist between these variables. Even though the repeated measures design limits the degree to which conclusions from the correlation model can be trusted, subsequent GEE analysis helps to mediate this limitation, and this design has the advantage of obtaining more data per subject than other studies, which will allow for more robust analyses as data from additional subjects is obtained.
